# Tibetan ‘wind’ and ‘wind’ illnesses: towards a multicultural approach to health and illness

**DOI:** 10.1016/j.shpsc.2010.10.005

**Published:** 2010-12

**Authors:** Ronit Yoeli-Tlalim

**Affiliations:** Wellcome Trust University Award Holder, Department of History, Goldsmiths, University of London, New Cross, London SE14 6NW

**Keywords:** Rlung, Wind, Tibetan medicine, Humours, Buddhism, Four Tantras, Mind-body, Culture-bound syndromes

## Abstract

This article discusses the Tibetan notion of *rlung*, usually translated as: ‘wind’, but perhaps better understood as a close equivalent of *pneuma* in the Greek tradition, or *qi* in the Chinese tradition. The article focuses on the way *rlung* provides a useful prism through which concepts of health, illness and disease may be observed in a cross-cultural perspective. An analysis of syndromes linked with *rlung* in a Tibetan cultural context illuminates some of the ways in which culture determines particular syndromes. The article raises a number of questions which are relevant for a more general multicultural approach to concepts of health, illness and disease.

The article argues that notions of *rlung*/*pneuma*/wind/ *qi* constitute a particularly interesting area for an exploration of culture-bound syndromes, as they reside in the meeting point between material and non-material, physical and mental, as well as the psychological, spiritual and religious. They are hence fundamental for a more cross-cultural approach to the mind-body problem.

The article also deals with the significance of history of medicine, particularly histories of medicine, which attempt to widen the scope of the traditional Eurocentric narrative of the history of medicine, in dealing with questions such as concepts of health and illness. Allowing alternative narratives—whether narratives of patients, other cultures or historical ones—can enhance our understanding of what health, illness and disease are. Discussing perceptions of the body as culturally defined is not only important from a philosophical or historical point of view, but also has important practical ramifications, which are particularly crucial in our global age.

Heinrich Jäschke, a nineteenth-century Moravian missionary to Ladakh, is mostly known amongst scholars of Tibet for his pioneering 1881 Tibetan–English Dictionary. Although originally his main task was to translate the Bible into Tibetan, his role in conveying Tibetan culture to the West was to become his main legacy. In his entry on ‘*rlung*’^1^—a fundamental concept of Tibetan medicine, commonly translated into English as ‘wind’—following his definition of the term, he added:‘These notions concerning *rluṅ* are one of the weakest points of Tibetan physiology and pathology’ (Bray, 1983).^2^ The Tibetan notion of *rlung* is indeed quite removed from western notions of physiology and pathology. As such, however, it provides a useful prism through which concepts of health, illness and disease may be observed in a cross-cultural perspective.

Philosophically, health has received less attention than disease (Murphy, 2008). While a philosophical discussion of the concept of health is beyond the scope of this article, it would be useful to consider the need for a multicultural approach to our understanding of health, illness and the relationship between them. Generally speaking, in the emerging field of philosophy of medicine, discussions of health and illness have remained within the western tradition and have not sufficiently taken into account Asian notions.^3^

An analysis of syndromes linked with ‘wind’ in a Tibetan cultural context illuminates some of the ways in which culture determines particular syndromes. In what follows, I raise a number of questions that are relevant for a more general multicultural approach to concepts of health, illness and disease.

Havi Carel’s book, *Illness: the cry of the flesh* has recently brought to the fore the discrepancies between disease as it is conceptualised by physicians and illness as it is experienced by patients (Carel, 2008). This philosophical inquiry may be further enriched by incorporating notions of illness as found in different cultures and across various historical periods. As suggested by Carel and Cooper, these different perspectives ought to be considered in a multidisciplinary manner, as well as informing health practitioners.^4^

Fundamental to this discussion is not only a conceptual analysis of illness, but also of health, as well as the relationship between the two. Is health to be viewed simply as the absence of illness? Or should health and illness be viewed as points on a continuum? What might be the value of approaching these questions from different historical and cross-cultural points of view?

At the basis of a multicultural approach to health, illness and disease lay different models of the body, as well as different ways of acquiring knowledge about the body. In this context it is worth noting the problematic usage of the term ‘anatomy’ in a cross-cultural perspective. The *Compact Oxford English Dictionary* defines ‘anatomy’, interestingly enough, both as ‘the scientific *study*^5^ of bodily structure’ and as ‘the bodily structure of a person, animal or plant’, that is, both the object of study and the study itself. Significant, here, is the etymology of the word, which is derived from the Greek word for ‘dissection’. The inherent bias towards acquiring knowledge about the body through dissection needs to be borne in mind when dealing with other medical systems that acquire their knowledge of some aspects of the body by other means—through introspection, for example.

Hence we note that intertwined with variant models of the body is the investigation into variant ways of acquiring ‘valid data’ about the body. In this context we ought to consider the extent to which modes of investigation are culture based and whether they lead to specific models and not others (Gyatso, 2004).^6^ An extensive body of literature has emerged recently, showing that perceptions of the ways in which the body is structured, perceptions of the ways it ought to be functioning—as well as the ways in which and purposes whereby these can be manipulated—are all culturally defined. These variant cultural viewpoints include religious notions, socio-political structures, and so on.^7^

One key aspect of alternative perceptions of the body is the notion of wind—a relatively close equivalent of *pneuma* in the Greek tradition and *qi* in the Chinese tradition. Important previous studies on this topic include work by Kuriyama,^8^ which has focused on Greek and Chinese notions, as well as two important cross-cultural collections (Kawakita, Sakai & Otsuka, 1995; Low & Hsu, 2007). In this paper, I raise a number of preliminary points regarding the notion of ‘winds’ as they are found in Tibetan medicine. When talking about ‘Tibetan medicine’, we generally speak about the medicine practised in areas which are under the influence of Tibetan culture. Namely, not only the political entity of the TAR (Tibetan Autonomous Region), which in the last half century has been a part of China, but also Ladakh, Nepal, Bhutan, Mongolia, Buryatia and parts of mainland China. Tibetan medicine is nowadays also practised in India (primarily among the Tibetan diaspora), as well as in the Russian Federation, Poland, Switzerland and other European countries.^9^

The following discussion serves as a contribution to what Kuriyama terms, ‘the problematic of breath’ (Kuriyama, 1995). As Kuriyama has suggested, ideas of *pneuma*/wind/*qi* reflect and define notions of the self. They also constitute perceptions of the body, and hence are central to any investigation of what defines health and illness.

## 1 The Tibetan notion of *rlung* (wind)

The Tibetan term *rlung* is rendered into English both as ‘wind’ and as ‘vital energy’, and has a host of meanings. *Rlung* also refers to outer wind, or breeze, as well as to the air.

The medical aspects of *rlung* are expounded in the *Four Treatises* or *Four Tantras* (*rGyud bzhi,* pronounced Gyu shi), a twelfth-century text and the *locus classicus* of Tibetan medicine, which is a Tibetan synthesis of Indian, Chinese and Greco-Arab concepts,^10^ grounded in Buddhist thinking, and which is still considered to be the key text of Tibetan medicine.^11^ Its basic view of health and illness is based on the notion of the three *nyes pa*s, usually translated as ‘humours’, but which literally mean ‘fault’ or ‘trouble’. These correspond to the Indian concept of the three *doṣa*, as they are termed in Sanskrit. In a medical context the three *nyes pa*s refer both to the potential causes of trouble or illness as well as to illness itself. As pointed out by Yonten Gyatso, the translation of *nyes pa*s into *humours* is problematic.^12^ The three *nyes pa*s are: *rlung* (wind), *mkhris pa* (bile) and *bad kan* (phlegm). The English terms given in brackets are the common translations of the Tibetan terms; they are not precise or unequivocal translations. What has been lost and gained through such translations requires its own investigation, which is beyond the scope of this paper. It is worth pointing out here, however, that the mere choice of translating the *nyes pa*s into *humours* not only conveys meanings but also *creates* meanings which are removed from their original ones.^13^

When out of balance, any of the three *nyes pas* can cause illness. Hence, there can be illnesses that are due to bile imbalance, phlegm imbalance or wind imbalance. Among the three *nyes pa*s, *rlung* is considered particularly important. It has a key dual role in both what in western terms would be defined as the psychic realm, and in the realm of somatic motility. According to the *Four Tantras*, the general functions of the winds are inhalation and exhalation, moving the limbs, as well as responsibility for different substances within the body. Wind is also responsible for all types of mental and verbal activities. The basic notion that the human body contains channels in which *rlung* resides is shared by Tibetan medicine and Tibetan tantric Buddhism.^14^ Leaving aside the numerous variations found in Buddhist tantric and medical texts,^15^ most descriptions agree that the human body has three main channels—one at the centre of the body, one on the left and one on the right—running from the head to the sexual organs. The Buddhist tantric practitioner investigates the movement of winds in these channels, and slowly acquires the ability to control them.

The *Four Tantras* discusses five types of wind.^16^

1. **Life-sustaining wind** resides at the centre of the body and generally governs the body/mind system.^17^ If the life-sustaining wind becomes disturbed, one may lose balance or consciousness. It can be the cause of various sorts of mental illnesses. Specific examples mentioned in the *Four Tantras* include: confusion, hearing sounds, visual hallucinations, and so on.
2. **Ascending wind** rules the throat and governs speech, memory, awareness, clarity of skin complexion and others. Malfunctions of this wind cause disorders of the upper part of the body, such as lung disorders, breathing difficulties, loss of voice, neck and shoulder pain, as well as headaches.
3. **Pervading wind**—according to Tibetan medicine—resides in the heart, but pervades the entire body. It governs body movements, makes blood and wind circulate throughout the body and nourishes it. Malfunctions of this wind manifest in loss of physical balance, tension, panic attacks, heart disorders, shoulder and back pain, blood circulation disorders and heart palpitations.
4. **Fire-like wind** resides in the stomach and intestines. Its main function is in regulating the digestive system. Psychologically, this wind manifests in desire for power, egotism, pride and greed. Its malfunctions cause chronic and acute digestive disorders, constipation, low metabolism, and so on.
5. **Descending wind** resides in the colon, bladder, reproductive organs and thighs, and governs the body below the navel. It functions downwards and controls waste evacuation, as well as semen and menstruation discharge. An imbalance of this wind could manifest as lower abdominal disorders, lower body blood circulation disorders, and particularly in psychological and emotional distress, such as jealousy, fear and worry.

### 1.1 Health according to Tibetan medicine

In Tibetan medicine, a sub-category of what may be termed ‘Buddhist medicine’, and particularly ‘tantric Buddhist medicine’, the knowledge and application of how to preserve one’s health is an important aspect of one’s spiritual path. The positive effects of physical health on one’s spiritual development are already recorded in the earliest Buddhist Pāli literature, such as the *Majjhimanikaya*.^18^ Already in early Buddhist writing health is seen as an individual’s finest possession and the difficulty of engaging in meditative practices if one’s body is in constant pain is also pointed out. Hence, from early on in Buddhism, knowledge of the body—as well as maintaining and restoring health—has been given soteriological significance. This has been particularly developed in tantric Buddhism, the Buddhist variant that reached Tibet. In this context, four aims are discussed: prevention of illness, cure of illness, securing longevity and attaining spiritual liberation. Tantric Buddhist soteriology elaborates the relationship between mind, body and spiritual liberation. Practically, this relationship manifests in the winds (rlung), and the way a practitioner learns to control them for the purpose of health maintenance, but ultimately, for the purpose of spiritual liberation.^19^

There are several chapters in the first and second of the *Four Tantras* that depict the body in its healthy condition, described in the Tibetan sources as an ‘unaltered state’. In this state, the three *nyes pa*s support and maintain the functions of the body. As long as the three *nyes pa*s, along with the seven body constituents and the three waste products are in a state of balance, the body will remain healthy. Any of the *nyes pa*s can be in a state of imbalance, which can be a state of excess, deficiency or disturbance. Factors which may cause the *nyes pa*s to be imbalanced could include inappropriate nutrition, lifestyle or behaviour. As explained by Yeshi Dhonden, the main difference between Tibetan and western medicine in this regard is that in Tibetan medicine advice regarding nutrition and lifestyle are specific to an individual’s constitution.^20^

The process of diagnosis in Tibetan medicine involves detailed questioning, observation and feeling the pulse.^21^ The questioning procedure will try to establish not only natural tendencies of the patient, but also immediate causes of any disorders. The physician will enquire about the patient’s diet, as well as lifestyle. Thus, for example, a stress-related lifestyle is seen as promoting *rlung* illnesses. The visual examination includes observing the tongue and the urine. The *Four Tantras* describe how a tongue of those with wind, bile or phlegm disorders would look, as well as how their urine would appear. Tibetan medical diagnosis also consists of an elaborate system of pulse diagnosis, taken at three different locations near each wrist.^22^

In the interpretation of all these diagnostic signs, the age of the patient will be taken into account, as well as the season, since natural tendencies change according to one’s age and according to season. These diagnostic methods require great skill and much practice, but are able to provide much important information about the patient.

According to the level of severity of the disorder, treatment would first involve a change of diet and recommendations regarding lifestyle. The next level would involve prescribing multi-component herbal medication, as well as massage, moxibustion,^23^ yogic and meditative practices, and finally various detoxifying treatments. In accordance with the basic theory, in both diagnosis and treatment the mind and the body are treated in tandem.

According to classical Tibetan medicine, a healthy body will result in long life, spiritual and material well-being, and ultimately has the potential of leading to the attainment of enlightenment. The link between health and spiritual attainment is illustrated in a set of medical paintings from the seventeenth century. Within this set of paintings, there is one painting dedicated to health and illness. The visual metaphor used in the painting is that of a tree: the trunk of health has twenty-five leaves which stand for conditions of health. The two flowers of this trunk are freedom from disease and longevity, and the two fruits are spiritual and material well-being and the obtainment of enlightenment.^24^ On the other hand, in cases of chronic or terminal illness, Buddhism provides spiritual and psychological comfort, which has been shown to be beneficial not only within a Buddhist context, but also in the West.^25^

In their natural healthy condition, people tend to be of either wind, bile or phlegm type, or more commonly of a combination of two of these (wind–bile; wind–phlegm; bile–phlegm). Illness arises when one or two of the *nyes pa*s are in excess or deficiency, or when their flow in the body is blocked. Such excesses or deficiencies arise due to a variety of conditions, including those relating to nutrition, mental state, time of year, climate, stage of life, and so on. Among the three *nyes pa*s, *rlung* has a more important status, as it is said to exacerbate whatever other imbalance the body has. Discussing the causes of illness, the second tantra says:

Distant causes (in turn) are general and specific. The body has been infinitely afflicted in various ways by humoural imbalances. Since it is not possible (here) to show every single cause, the general causes of all disease will be expounded. The sole cause of all disease is said to be ignorance due to lack of understanding of the meaning of selflessness […]

Specific causes: from ignorance arise the three poisons of attachment, hatred and closed-mindedness whence are produced in turn the humours wind, bile and phlegm.

Proximate causes

Undisturbed wind, bile and phlegm are the causes of disease whilst disturbed, imbalanced humours are the nature of disease. They harm the body and life, and give rise to suffering […].^26^

Causes of illness, according to the *Four Tantras*, are divided into primary causes (*rgyu*) and secondary causes (*rkyen*). The primary causes can be viewed as basic potentials for illness, while the secondary causes are more immediate, concrete ones. The primary causes are the Three Poisons (*dug gsum*), central to Buddhist thought: attachment or greed (*‘dod chags*), ignorance or mental darkness (*gti mug*) and anger (*zhe sdang*). The three poisons are linked with the three *nyes pa*s: attachment and greed are linked with *rlung* (wind); ignorance/mental darkness with *bad kan* (phlegm); anger with *mkhris pa* (bile). The secondary causes of wind illness are described in the second chapter of the third tantra as follows: relying excessively on bitter and rough (non-nourishing) foods; being overtired; engaging in strenuous or intense physical and verbal activities (especially on an empty stomach); losing much blood; being exposed to cold wind; excessive weeping; intense grief (mya ngan,—also: worry, despair, distress); repressing bodily impulses and forcing bowels by pressure.^27^

In Tibetan medicine, diverse symptoms—such as memory weakness, constipation, dizziness, shivering and certain skin disorders—can all be diagnosed as resulting from excess of wind. The complex method of diagnosis establishes the underlying cause of ambiguous symptoms, such as skin disorders. Once these symptoms are diagnosed as resulting from excess of wind, they could all be, in principle, treated in the same way. This is, obviously, fundamentally different from a biomedical approach.

Tantric yogis engage in a variety of meditative practices in order to be able to know and control these winds. This, in turn, enables them to control the five consciousnesses, as well as mental consciousness. The winds all move in numerous channels across the body, but there are three main channels: left, right and centre. The aim of tantric yoga is to bring about a concentration of the winds. Through these meditative practices, tantric yogis aim to refine their physical and subtle body. The subtle body consists of channels (known in Sanskrit as *nadi*, and in Tibetan as *rtsa*), in which the winds flow, as well as points of intersection (*chakra*) at which these channels come together.^28^

This brings us back to the point raised earlier about the way a method of investigation, in this case meditation, leads to the nature of knowledge attained. In the case of Buddhism and Buddhist-derived medical systems like the Tibetan one, the method is also intertwined with the goal. The various yogic exercises that involve different bodily postures and breathing techniques are not only used for investigating the inner workings of the body, but also for maintaining one’s health; restoring one’s health, if it has become unbalanced; and, ultimately, reaching spiritual attainment.^29^ The ultimate goal is spiritual attainment, but balancing one’s winds as a way of maintaining one’s health is considered important as well.

Knowledge of these notions of winds as they are discussed in the *Four Tantras* and Buddhist literature is presently not confined to Tibetan physicians and monks, but is common among Tibetan laity.^30^ As discussed in a number of anthropological works, Tibetans commonly know what constitutional ‘type’ they are, and engage at various levels in ‘self-diagnosis’. They would refer to ‘having wind’ or ‘high wind’ (*rlung mtho po*) to indicate a state of emotional distress. As delineated in the *Four Tantras*, people ascribe wind conditions to physical, mental and emotional factors. Jacobson’s reports from his fieldwork mention exhausting physical work, exposure to bad weather and reliance on poor nutrition as causes of physical ‘wind’. Additionally, Jacobson’s informants reported too much worry, separation from loved ones, and exhausting intellectual work as causes of ‘wind’ problems.^31^ Tibetan refugees linked many of these wind conditions with their difficult political and personal circumstances, in what has been termed ‘diseases of exile’.^32^

A further illustration of the way *rlung* illnesses most readily lend themselves to cultural interpretation is found in the anthropological work of Colin Millard, who has studied approaches to mental illness within a clinic of Tibetan medicine (Tara Institute) in the United Kingdom and in a Tibetan medical school in Dhorpatan (Nepal). Discussing the differences between the ways mental illnesses are constructed by patients in these two settings, Millard addresses a major debate in transcultural psychiatric studies regarding whether certain features of psychiatric experience are universal or whether they are entirely framed by the culture in which they occur. Analysing the divergence between these two settings, he shows how culture defines the general epistemology and etiology of an individual’s condition (Millard, 2007).

Tibetan notions of wind as they are portrayed in medical and Buddhist literature determine a relationship between physical and mental aspects of the body, as well as between the individual and her/his environmental conditions. The anthropological data show that these relationships are still at the fore for contemporary Tibetans and determine the ways in which they construct their concepts of illness and health. Rather than a dichotomous view of health and illness as mutually exclusive, the Tibetan model presents a continuum, with critical illness at one end and spiritual attainment at the other, and in which ordinary ‘health’ and ‘illness’ are located somewhere in between.

Exposing the significance of underlying cultural assumptions in the construction of medical models of the body may help us see that these medical models—as Leon Eisenberg had already pointed out in the 1970s—are ‘indispensable but hazardous because they can be mistaken for reality itself rather than as but one way of organising that reality’ (Eisenberg, 1977). The dual meaning of the term ‘anatomy’, discussed earlier, as both the ‘scientific study of bodily structure’ and ‘bodily structure’ serves as an illumination of this point.

It is worth emphasising the usefulness of the history of medicine in this endeavour, and particularly a more cross-cultural type of history of medicine, attempting to widen the scope of the traditional Eurocentric narrative of the history of medicine. Allowing alternative narratives—whether narratives of patients, other cultures or historical ones—can enhance our understanding of what health, illness and disease are. As Sheldon Watts has commented, during the last 5,000 years, each cultural grouping on the planet has had their own way of explaining and treating illness and disease. ‘Disease history’, as Watts points out, ‘thus alerts us to the diversity of the human experience’. Furthermore, ‘[t]he road to understanding the totality of the human experience (world history) lies through accepting the need for a pluralistic approach”.^33^ Cross-cultural historical analyses uncover multiple narratives on the body, on health and illness, allowing a better understanding of the interdependence between certain cultural perceptions and notions of health and illness. In order to develop these analyses, history of medicine, or rather histories of medicine, medical anthropology and philosophy of medicine are all vital to each other.

### 1.2 Practical implications

Discussing perceptions of the body as culturally defined is not only important from a philosophical or historical point of view, but also has practical ramifications. Within this scope, medical anthropology, for example, has been increasingly influential in fields such as mental health. There is an extensive body of literature dealing with the notion that mental health and mental illness need to be addressed in a cross-cultural perspective.^34^

Similarly, the cross-cultural approach to health and illness is central to public health. It is worthwhile mentioning here the World Health Organization (WHO)’s definition of health, adopted in 1946: ‘Health is a state of complete physical, mental and social well-being and not merely the absence of disease or infirmity’.^35^ Although this definition has been criticised,^36^ it is worth observing its rather broad approach. Commenting on the way in which this definition was constructed, with its post-war immediacy and ad hoc cross-cultural nature, Dr Szeming Sze, a Chinese doctor and one of founders of WHO, writes:

I think there were three of us—Dr Brock Chisholm from Canada (who became the first Director-General of WHO), Dr Gregorio Bermann from Argentina, and myself; it was a pleasant little group and we had some interesting academic discussions. Chisholm, being a psychiatrist, wanted to mention mental health, and I thought we should put in something that emphasized the importance of the preventive side of health. That’s how we came up with the wording in the Constitution that defines health as not merely the absence of illness.^37^

Although we can merely speculate, it seems that the insistence on preventive medicine by Sze is derived from an Asian point of view.

Since the adoption of this definition, the openness to the existence of multiple narratives describing the body, as well as to the existence of alternative approaches to health and illness, has been growing slowly. The interest in Tibetan Buddhism and medicine in the West in recent years has focused on the meeting between these Tibetan traditions and neuroscience, mind–body medicine and psychotherapy, among others.^38^

In various fields in academia, cross-cultural perspectives are increasingly found in some fields of humanities, demonstrated, for example by the rise of global history (or world history) as a field of study and research. With the development of this perspective, narratives from the non-Western world are slowly brought to the fore.^39^ These processes are also intertwined with the permeating effects of challenging the validity of any single narrative, increasingly allowing alternative narratives to find their place.

Although addressing the question of efficacy still poses an enormous methodological problem, there is by now a large body of studies which reveal the benefits of Tibetan medicine, particularly as a complementary medicine. Research has been conducted on the efficacy of Tibetan medical treatments in relation to a wide range of conditions, such as cardiology, pulmonology, gastrointestinal medicine, infectious diseases, oncology, psychiatry, rheumatology, urology, rehabilitation medicine, dermatology and palliative care.^40^ These come in addition to an ever-growing body of literature which has discussed the benefits of Tibetan medical practices in an area gaining increasing importance in health care: well-being and caring for the aged.^41^ Due to its comprehensive approach to the mind and body, as briefly described earlier in the overview of *rlung,* Tibetan medicine and, more generally, various aspects of Buddhist-based medicine, is gaining a growing place within preventive and behavioural medicine. A leading figure within this stream is Jon Kabat-Zinn, who has been researching and teaching mindfulness meditation to people with chronic pain, stress-related disorders and breast cancer, among others. Kabat-Zinn has also conducted important work on the cost-effectiveness of these kinds of interventions.

Tibetan medical theory is also able to provide alternative paradigms to those found in mainstream biomedicine, by virtue of its greater consideration of patients’ own experience of their illness; consideration of patients’ psychological and sociological contexts as possible determinants of health; as well as the vital importance of prescribing particular foods to particular people as an effective method of preventive health as well as for the treatment of mild disorders. Many of these are slowly finding their way into various forms of alternative and mainstream medicine. As such they provide a fruitful alternative to problematic aspects of mainstream biomedicine, some of which have been delineated by Carel^42^ and Eisenberg.^43^

Yet, as Joseph Loizzo—a medical doctor who has been engaged with the integration of Tibetan medical knowledge into western practice for many years now—has pointed out, two causes have so far hindered the great potential for further collaborations and utilizations of this type. First, the West has only recently gained access to the Tibetan Buddhist texts which provide some of the theoretical framework for Tibetan medicine. An even greater lack becomes evident when dealing with Tibetan medical texts themselves. There is still no translation into any European language of the *Four Tantras*, the text that is the basis of Tibetan medicine.^44^ Related to these factors are the misconceptions and the various ‘methodological mismatches’, as Loizzo, Blackhall & Rapgay term them, when trying to apply Tibetan medical concepts to western scientific research. In spite of the abundance of methodological hurdles, Buddhist medical science has already shown its potential, as they suggest, to serve as an ‘extraordinary science’, improving ‘normal science’ in Kuhn’s terms.

If one attempts to sum up the collaborative work which has been conducted between clinicians and traditional Tibetan doctors so far, we could say that the first decades were focused on the question—to put it crudely—‘Can we talk to each other?’ The next stage has focused on the question: ‘Can we learn from each other?’ With overall positive answers to both, collaborative work in recent years has been increasingly focused on finding appropriate methodologies.

In the current state of affairs, philosophers of medicine and science have an important role to play in suggesting further advances in overcoming these ‘methodological mismatches’. In an illuminating analysis, Loizzo, Blackhall & Rapgay recently suggested the adoption of a relativist view of complimentary science for complimentary medicine. They propose studying Tibetan traditional medicine, like other Eurasian traditional systems, as:

[…] alternate theoretical and practical frames of reference for approaching problems to which current biomedical models and methods may be ill-suited. In particular, we see them as applicable to the family of problems where the mechanistic models, invasive diagnostics, and manipulative treatments of conventional biomedicine have a high cost and limited benefit, and where better, more cost-effective outcomes may result from models and methods that help patients change their mindset, behaviour, and lifestyle.^45^

One possible aid in developing alternate theoretical frames of reference is a recent development in biology: systems biology. Systems biology is a novel approach to analysing biological complexities, which studies organisms as ‘integrated and interacting networks of genes, proteins and biochemical reactions which give rise to life’.^46^ Systems biology has been developed in the last decade, particularly since the year 2000 by Prof. Jan van der Greef and others, to serve as the basis for predictive, preventive and personalised medicine, acting as a bridge between traditional Asian medicine and western medicine. Its terminology and methodology—all originating from western bioscience—enable numerous fruitful interactions between Asian medicine and western bioscience. Such is, for example, the development of scientific methodology that can be applied to investigate the multi-target approach of Tibetan and other Asian and traditional medical systems. The tools developed by van der Greef of ‘biomarkers’ (‘fingerprints’) are able to provide a more holistic picture of multi-parametric pharmacological interventions used in Tibetan or Chinese medicine. The practical implications of these in crucial areas such as global health are only now beginning to be discussed.^47^

What is clear to many philosophers of science and medicine—that concepts of health and illness are – at least to some extent – culturally defined, has yet to be elaborated with respect to their potential impact on the various health professions. Further work in this area as well as additional development relating to the difficult question of methodology—and their dissemination among the scientific and medical professions—would allow further research into areas in which Tibetan medicine and other traditional Asian medical systems could fruitfully augment biomedicine.

All of this, however, is dependent on the survival of traditional Tibetan medicine. There is not only a need for more research into such medicine, but also a need to establish financial support for the preservation of this rich tradition.^48^

As Leon Eisenberg put it, the ‘chastening discovery that other theories of disease, and practices based on them, can produce benefit helps to free us from medical ethnocentrism’.^49^ At the basis of this chastening discovery is the acknowledgement that different cultures use different notions of the body and different notions of the interactions between body and mind, and hence they also define concepts of syndromes and illness differently. If sent to Tibet today, Heinrich Jäschke would probably not have been as dismissive as he was.

## Footnotes

1 When providing Tibetan words, I use the Wylie system of transliteration. ‘rlung’ is pronounced: loong.

2 For more on H. A. Jaschke, see Bray (1983).

3 Standard work on this topic includes the work by Christopher Boorse, such as: Boorse (1975, 1977), Reznek (1987), Cooper (2002).

4 This is a key objective in their multidisciplinary network “The Concepts of Health, Illness and Disease”. See: http://www.uwe.ac.uk/hlss/courses/philosophy/ahrc_chid_network.shtml, last accessed 22 February 2010.

5 My emphasis.

6 For an illuminating essay on a Tibetan aspect of this question, see Gyatso (2004).

7 Some of the important work in this direction includes: Kuriyama (1999), Lloyd & Sivin (2002), Unschuld (2009), Watts (2003), Feher, Naddaff & Tazi (1989), Fraser & Greco (2005).

8 Kuriyama (1999).

9 For the fascinating account on how Tibetan medicine became a prominent form of medicine in the court of the Russian Tsar and the Russian army during the nineteenth and early twentieth centuries, see Saxer (2004).

10 On the connection between Tibetan and Greco-Arab medicine, see Martin (in press); and (my own) Yoeli-Tlalim (in press).

11 For an English translation of the first two tantras, see Clark (1995). For an overview of Tibetan medicine by one of its most well known practitioners, see Dhonden (2000).

12 On the inadequacy of the translation of *nyes pa* into humours, see Gyatso (2006).

13 It also appears that many of the claims pointing out the affinity between the Greek and the Indian medical systems with regard to the *doṣa*/humour system are ultimately drawn from the translation choice of ‘humour’ for *doṣa*, though this requires further research.

14 What follows is only a very rudimentary explanation. For more detailed expositions on channels and rlung in tantric Buddhism, see: Cozort (1986); for a discussion of the channels in the Ka¯lacakra, see Wallace (2001) particularly pp. 101–106; and Kilty (Trans.) (2009), White (2000). For further details and visual illustrations of some of the channels which form aspects of the subtle body in the Tibetan medical tradition, see Parfonovitch, Meyer, & Dorje (1992), Medical painting no. 12, Vol. 1 pp. 39–40, Vol. 2 pp. 195–196; Garrett & Adams (2008), Gerke (2007).

15 These are discussed by Garrett & Adams (2008).

16 Another set of five winds are those associated with what in Buddhist terms is called the Five Consciousnesses—that of the eye, ear, nose, tongue and body. Consciousnesses are said to ‘ride’ on winds. The following summary is based on teachings by Pasang Yonten.

17 The general topic of the body–mind issue from a Buddhist perspective—as well as its relationship with western notions—is an enormous question, worthy of more collaborative work between western philosophers and Buddhologists. For some of the contributions on this topic, see, for example: Harvey (1993), Griffiths (1986), Yuasa (1987).

18 The following section is based on Wallace (2009).

19 For a very useful overview, see, Skorupski (1999).

20 See Dhonden (2000).

21 For a detailed account on diagnosis in Tibetan medicine, see Meyer (1992).

22 This Tibetan pulse diagnosis system has some similarities with and some differences from Chinese pulse analysis. For a detailed study, see Meyer (1990).

23 Moxibustion, also common in Chinese medicine, is a system of heating specific points of the body in order to treat various disorders.

24 See Parfonovitch, Meyer, & Dorje (1992). Medical painting no. 2, Vol. 1 pp. 19–20, Vol. 2 pp. 175–176.

25 See, for example, Cohen, Warneke, Fouladi, Rodriguez, Chaoul-Reich (2004), Chaoul (2007). There is extensive literature on the use of mindfulness for facing terminal and other illnesses. See, for example, Kabat-Zinn (1998).

26 Clark (Trans) 1995, pp. 75–76.

27 *Four Tantras*, Third tantra, Chap. 2.

28 For a discussion of the subtle body and practices associated with it, see Samuel (2008), especially chap. 11.

29 For a detailed account, see Wallace (2008).

30 The following summary of notions of winds among Tibetan laity is based on work carried out by Eric Jacobson. See Jacobson (2007). See also Samuel (2007).

31 Jacobson (2007).

32 For an extensive treatment of Tibetan ‘diseases of exile’, see Prost (2007).

33 Watts (2003), p. 1.

34 See, for example, the journal Culture, Medicine and Psychiatry. See also Helman (2000).

35 Preamble to the Constitution of the World Health Organization as adopted by the International Health Conference, New York, 19–22 June 1946; signed on 22 July 1946 by the representatives of 61 States (Official Records of the World Health Organization, no. 2, p. 100) and entered into force on 7 April 1948. WHO website: www.who.int/about/definition/en/print.html last accessed October 19, 2010.

36 See, for example, the criticism by Alejandro Jadad and Laura O’Grady in an editorial in the British Medical Journal (Jadad & O’Grady, 2008). Proposing to experiment with what they describe as ‘a new form of civil society participation, enabled by the growing use of the internet, mobile phones, and related information and communication technologies’, they have established a blog on which anyone with internet access (and English proficiency, it should be added) can contribute her/his view of the definition of health.

37 See http://whqlibdoc.who.int/analytics/WHForum_1988_9(1)_29-34.pdf, last accessed 22 February 2010.

38 The relevant literature is extensive, but see, for example, Kabat-Zinn (1998), Goleman (2003a, 2003b), Loizzo & Blackhall (1998), Loizzo (2000). See also the forthcoming (2011) issue of Contemporary Buddhism dedicated to applications of mindful-based therapies.

39 In the field of history of science, see, for example, Bala (2007). Slower to incorporate a more non-Eurocentric view is the field of philosophy of science based on eastern thought. Innovative, interesting work in this respect has been published by Evan Thompson. See for example his co-authored Rosch et al. (1991) and Dreyfus and Thompson (2007).

40 Many of these studies are discussed in Loizzo, Blackhall & Rapgay (2009). The entire volume in which their article appeared is dedicated to laying the ground for what the editor, William Bushell of MIT, terms a ‘contemporary cosmopolitan science’, in which Tibetan medicine could be further explored in western contexts.

41 See the numerous publications of the Mind and Life Institute, such as: Begley (2007), Goleman (2003a), Goleman (Ed.) (2003b). Regarding aging, see the collection of articles in: *Longevity, Regeneration and Optimal Health: Annals of the New York Academy of Science*, 2009, Vol. 1172.

42 Carel (2008).

43 Eisenberg (1977), pp. 9–23.

44 See note xi.

45 Loizzo, Blackhall, & Rapgay (2009), p. 225.

46 See www.systemsbiology.org, last accessed 20 April 2010.

47 The recent international symposium of the Institute for System Biology, titled: ‘Systems Biology & Global Health’ (18–19 April 2010) was dedicated to exploring many of these questions.

48 Many of the crucial difficulties that Tibetan medicine is facing are discussed in Schrempf (2007).

49 Eisenberg (1977), p. 21.
